# Priorities, actions and risks in the COVID-19 pandemic: a flash SoMe survey among surgical oncologists

**DOI:** 10.1515/pp-2020-0142

**Published:** 2021-01-25

**Authors:** Delia Cortés-Guiral, Olivia Sgarbura, Mohammad Alyami, Kazuhiro Yoshida, Yuichiro Doki, Hironori Ishigami, Fabian Grass, Martin Hübner

**Affiliations:** Department of General Surgery and Surgical Oncology, King Khalid Hospital, Najran, Saudi Arabia; Department of Surgical Oncology, Cancer Institute Montpellier (ICM), Montpellier, France; Department of Surgical Oncology, Gifu University School of Medicine, Gifu, Japan; Department of Gastroenterological Surgery, Graduate School of Medicine, Osaka University, Suita, Osaka, Japan; Department of Surgical Oncology, The University of Tokyo, Tokyo, Japan; Division of Colon and Rectal Surgery, Mayo Clinic, Rochester, MN, USA; Department of Visceral Surgery, Lausanne University Hospital CHUV, University of Lausanne (UNIL), Lausanne, Switzerland; University of Montpellier, Montpellier, France

**Keywords:** COVID-19, priorities, surgical oncology

## Abstract

**Objectives:**

Corona virus-induced disease 19 (COVID-19) pandemic has globally affected the surgical treatment of cancer patients and has challenged the ethical principles of surgical oncologists around the world. Not only treatment but also diagnosis and follow-up have been disrupted.

**Methods:**

An online survey was sent through Twitter and by the surgical societies worldwide. The survey consisted of 29 closed-ended questions and was conducted over a period of 24 days beginning in March 26, 2020.

**Results:**

Overall, 394 surgical oncologists from 41 different countries answered the questionnaire. The predominant guiding principle was “saving lives” 240 (62%), and the different aspects of lock-down found hence large support (mean 7.1–9.3 out of 10). Shut-down of elective surgery and modification of cancer care found a mean support of 7.0 ± 3.0 and 5.8 ± 3.1, respectively. Modification of cancer care longer than two weeks was considered unacceptable to 114 (29%) responders. Hundred and fifty six (40%) and 138 (36%) expect “return to normal” beyond six months for surgical practice and cancer care, respectively.

**Conclusions:**

Surgical oncologists show strong and long-lasting support for lock-down measures aiming to save lives. The impact of the pandemic on surgical oncology is perceived controversially, but the majority was forced already now to accept what is inacceptable for many of their colleagues.

## Introduction

Corona virus-induced disease 19 (COVID-19) pandemic changed our professional and private lives [1], [2], [3] since a brief report to WHO China of few cases of respiratory pneumonia of unknown origin at the end of December 2019 [4] turned into a declaration of Public Health Emergency of International Concern (PHEIC) on January 30 [5]. Radical measures needed to be taken in most countries to contain infectious spread and to increase or shift resources from most domains to emergency units and intensive care aiming to avoid to increase the surge capacity of health care systems and to minimize the number of avoidable COVID-19 deaths [6], [7], [8], [9], [10], [11], [12], [13], [14], [15], [16].

The general principle of patient care “*primum not nocere*” was challenged in COVID-19 times, as some of these measures required to limit surgical service and to delay or modify cancer care with yet unknown consequences for the concerned patients [17], [18], [19], [20], [21].

The aim of this survey was to study the surgical oncologist’s view on priorities, risks and measures during the COVID-19 pandemic with special focus on the impact on surgical oncology.

## Materials and methods

The present study is a flash survey among surgical oncologists diffused by Social Media (SoMe). The questionnaire consisted of 27 closed-ended questions on demographics (n=5), (A) guiding principles (n=2), (B) measures of containment (n=10), (C) shifting of resources, current management (n=6) and (D) perspective (n=4) (Supplementary Material, Appendix p 1). The intuitive online questionnaire (Google Docs^®^ application, 2012, Mountain View, California, USA) could be answered from any connected device (mobile phone, personal computer, tablet and laptop) and took a median of 7 min to be completed.

The survey was launched online on March 26, 2020 while the pandemic spread exponentially [22], [23], [24] and was closed on April 19, when death tolls peaked in the United States [25], [26], [27]. Twitter is the most engaged social media application for physicians around the world [28, 29] and was therefore chosen as platform to launch the survey. The survey was diffused through the SoMe4Peritoneum account @SPeritoneum, SoMe4Surgery @me4_so and SoMe4HPB @hpb_so (Twitter official accounts from SoMe4Surgery communities specifically focused on surgical oncology and peritoneal surface malignancies) and the private account of one of the surgical oncologists in charge of this community (@DeliaCortesGuir). Concurrently the survey was endorsed and distributed by the following surgical societies: ACS (American College of Surgeons), ESSO (European Society of Surgical Oncology), JSCO (Japan Society Clinical Oncology), RENAPE (French Registry for Rare Peritoneal Tumors), SEOQ (Sociedad Española de Oncología Quirúrgica) and SSCRS (Saudi Society of Colon and Rectal Surgery).

Plain descriptive statistics were used to present the results of the current survey and the frequencies were reported as raw numbers and percentages. Median and mean values were calculated for discrete and continuous variables, respectively. Data analysis was performed with IBM© SPSS^®^ Statistics Subscription (IBM Inc., Armonk, NY, USA). Discrete variables were compared with Wilcoxon test, categorical variables were compared with χ^2^-test and correlations were performed with Pearson and Spearman tests. A two-sided alpha of 0.05 was used to indicate statistical significance.

## Results

In the 24 day time period, 394 surgical oncologists from 41 different countries answered the questionnaire. Complete demographics are provided as Supplementary Material, Appendix p. 6. Briefly, 268 (68%) responders worked in academic institutions or dedicated cancer centers. Most responders were consultants 297 (76%) and had at least one subspecialty 334 (85%).

### Guiding principles

**Figure 1:**
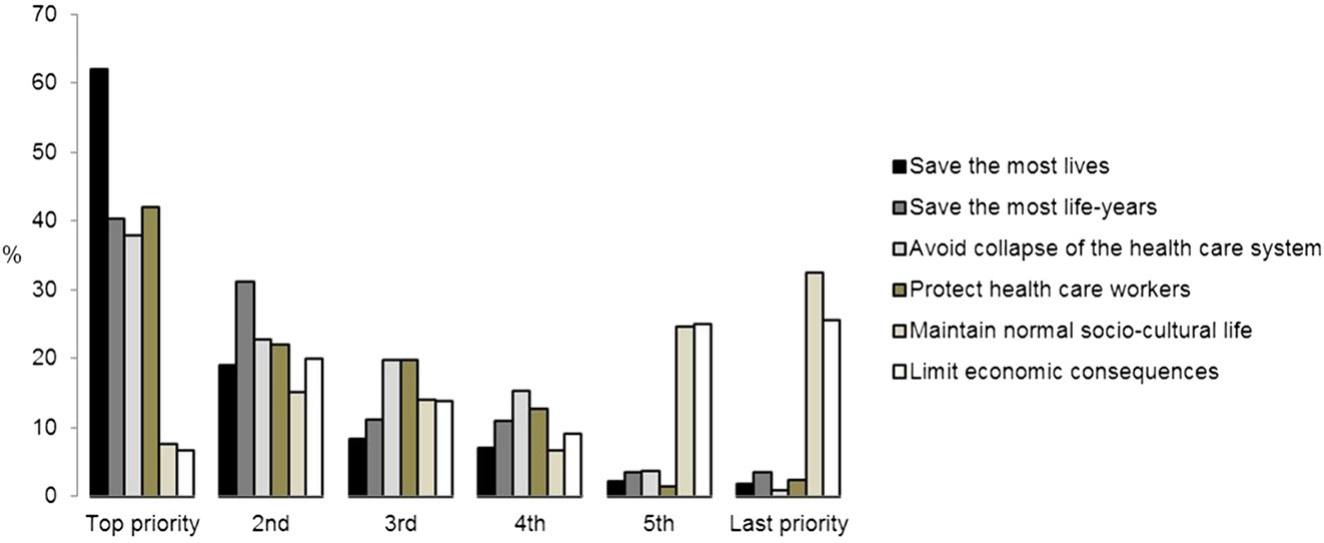
Guiding principles during COVID-19 pandemic. Schematic representation of guiding principles during COVID-19 pandemic ranked by priority.

Figure 1 displays the wide variation of priorities among surgical oncologists concerning pertinent guiding principles. As a pattern, three different levels of priority aims could be identified: 1. Saving lives, 2. Saving life-years, protection of health care system and workers, 3. Maintaining normal life and limit economic consequences. Of note, two-thirds acknowledged that these declared priorities were likely to change in the future course of the pandemic.

- Measures of containment

**Figure 2:**
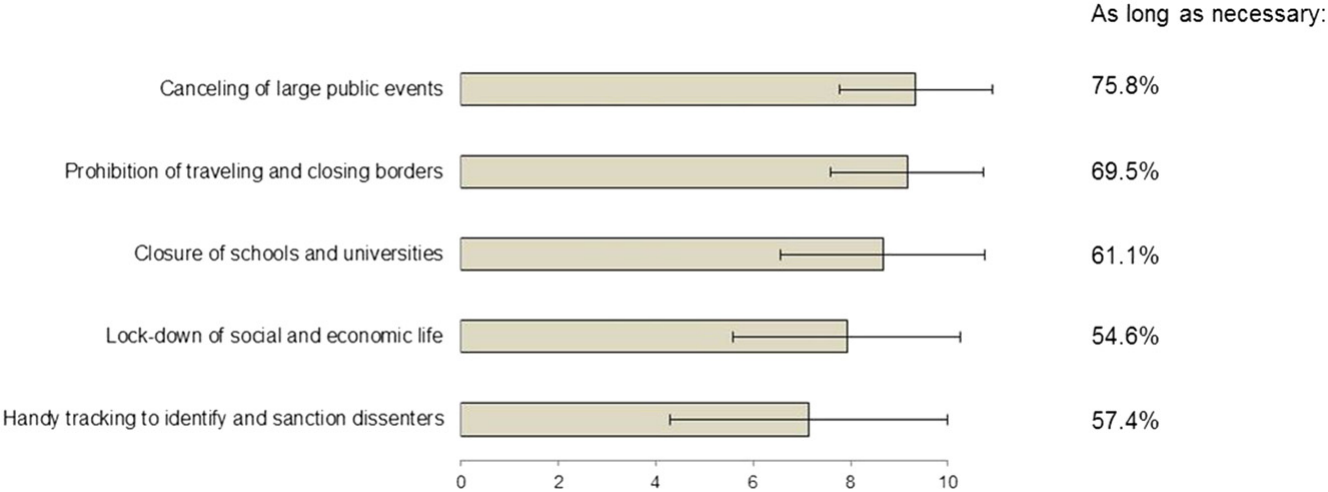
Measures of containment during COVID-19 pandemic: support and acceptable duration. Support for measures of containment during COVID-19 pandemic rated from 0 (no support) to 10 (total support). Displayed are means and standard deviation. The majority of responders supported containment as long as necessary, as indicated by percentages for each containment measure.

Restrictive measures found variable support by the responders and were considered inevitable by 298 (76%) panelists for canceling of large public events, 244 (62%) for limitation of travel, 221 (56%) for closure of schools, 140 (36%) for lock-down and 107 (28%) for handy-tracking. Mean support for these measures of containment is displayed in Figure 2. A variable majority (55–76%) supported all of these measures for “as long as necessary”.

B. Shifting resources

**Figure 3:**
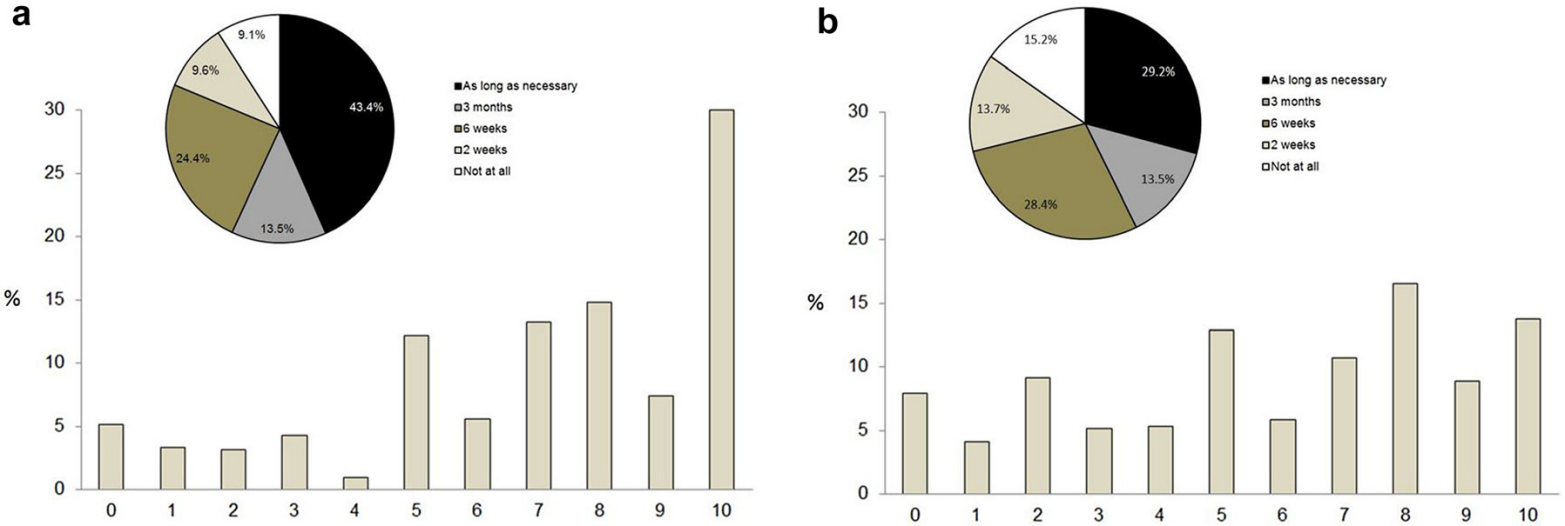
Acceptance of shifting of resources during COVID-19 pandemic. (A) Shut-down of elective surgery program. (B) Deferring or modification of cancer care. Acceptance of shifting of resources for (A) shut-down of elective surgery program and (B) deferring or modification of cancer care rated from 0 (no support) to 10 (total support). Pie charts represent acceptable time lines for shifting of resources.

**Figure 4:**
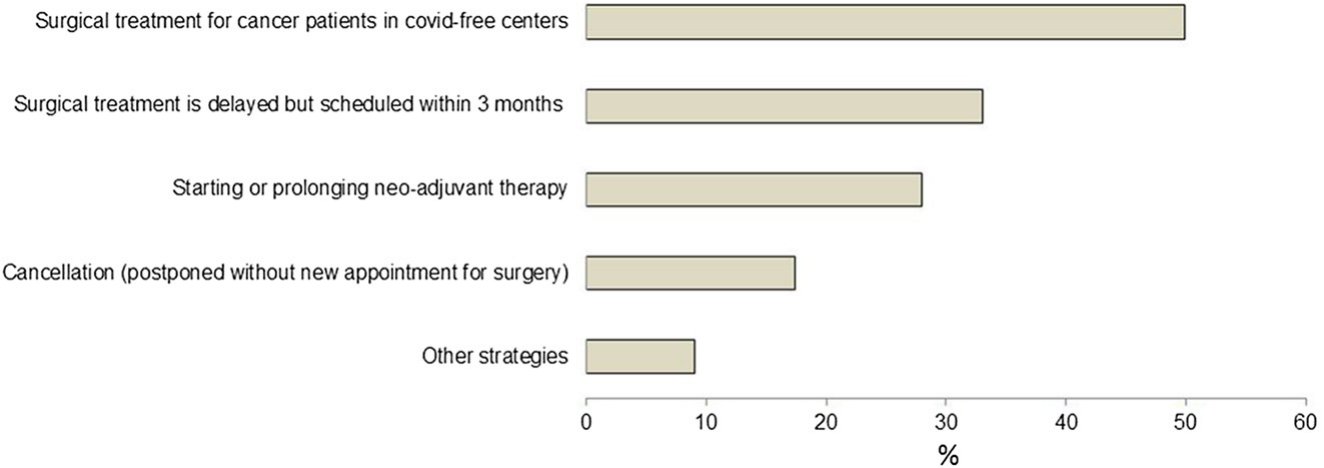
Changes in management of cancer patients during the COVID-19 pandemic.

Large heterogeneity was encountered with regards to the shutdown of elective surgery program (mean support 7.0 ± 3.0) and modification of cancer care (Figure 3A, B). The latter found little support by surgical oncologists (mean support 5.8 ± 3.1). Interestingly 114 (29%) of responders judged modification of cancer care to be inacceptable at all or for >two weeks, while 115 (29%) could accept this profound paradigm shift for “as long as necessary”. Further, 258 (66%) declared to have changed their strategy already as detailed in Figure 4.

C. Perspectives

Estimated duration until “return to normal” for surgical practice, cancer care, medical meetings and social life are summarized in Table 1. Of note, return to normal surgical practice and cancer care was not expected to occur before six months of time by 156 (40%) and 138 (36%) of the surveyed surgical oncologists, respectively.

D. Intercultural variations

**Table 1:** Estimated duration of COVID-19 crisis: “Back to normal”.

Normality in …	Six weeks	Three months	Six months	One year +
Surgical practice	24%	***37*%**	28%	12%
Cancer care	28%	***37*%**	25%	11%
Meetings	7%	21%	***46*%**	26%
Social life	8%	31%	***37*%**	24%

Highest proportion is displayed in bold.

The results of the main group of respondents (Japan) were compared to the rest of the participants. Of note, a significantly lower rate of surgeons needed to change their strategies of cancer care (31 vs. 88%, p<0.0001) but a similar number estimated that they might change their priorities in the future (62 vs. 70% in the rest of the group, p = not significant [NS]). The ranking of priorities was similar in the two populations. Global support for the measures of containment was significantly lower in the Japanese population (8 vs. 9.2, p<0.0001) and the difference was confirmed for every category.

Support for deferring cancer care was significantly lower than for all other measures of containment in the entire population (5.8 vs 8.7; p<0.0001). The support was lower in the Japanese subgroup than in the rest of the respondents (5.3 vs 6.3; p<0.0001).

## Discussion

The present flash survey depicts the current view of surgical oncologists on priorities, measures and their impact on surgical care. The top priority guiding principle “to safe lives” leads currently to wide acceptance of restrictive measures despite the perceived negative impact on cancer care. While there is reluctance to delay or modify surgical oncology, this change has widely taken place already and it is expected to last for at least six months.

Fair allocation of resources in situation of scarcity has been long discussed in different contexts [30, 31] and it is nowadays accepted that the four leading principles are: maximizing benefits of scarce resources, treating people equally, promoting and rewarding instrumental value and giving priority to the worst off. In this survey, “saving most lives” came up as a top priority for most respondents while “saving most life-years” appeared more frequently in the second position but was almost as frequently cited as the third or even fourth priority. These two principles are considered as the equivalent of a consensus in bioethics [30], [31], [32] and, although some heterogeneity was noted, surgical oncologists recognized them as such.

Although the support of surgical oncologists for different containment measures was variable, cancelation of public events, limitations of travel and closure of schools obtained a moderate to large consensus (more than 60%) just as a similar rate was encountered in support of these measures “as long as necessary”. These results are consistent with the fact that surgical oncologists are caregivers placing healthcare in the top of the societal prime concern, particularly in pandemic context. Given the large range of nationalities of the respondents of these surveys and the share of 38% of Japanese oncologists, these results imply that professional focus outreaches the cultural specificity in this issue. Since the first report indicating a significantly higher rate of COVID-19 infections in cancer patients [20], several national and international guidelines proposed recommendations concerning the potential deferral mechanisms in cancer care and targeted pathologies [33], [34], [35], [36]. In the light of these recommendations, some authors [37, 38] analyzed big data in a tentative to calculate a safe postponement period that turned out to be a median of three weeks since specialist consultation and six weeks since diagnosis for cancers treated with surgery first. In the present survey, two-thirds of the respondents acknowledge to have changed their cancer care strategies during the COVID-19 pandemic. The acceptable deferring period for cancer care was the item with the highest heterogeneity in this study. Of note, one third of the respondents considered that deferral was unacceptable or acceptable for a maximum of two weeks while another third accepted it for “as long as necessary”. These results are highly suggestive of a potential conflict faced by surgical oncologists during the present sanitary situations as the traditional pressure put on cancer care systems to be highly responsive in the management of the disease collides with present ambiguous recommendations from scientific societies and scant evidence-based data.

Even in normal times, rationing of the resources and, potentially, of healthcare is unavoidable [39]. However, it has been showed before that practitioners, including ICU practitioners that are exposed to that selection on a regular basis, are not always aware of the choices they are making [39]. Furthermore, the individual physician should not be faced with the terrible task of performing the selection in isolation [30]. The current situation is sometimes exposing the surgical oncologists to making that choice on their own or together with their colleagues in local multidisciplinary tumor boards which questions their preparation to facing bioethical dilemmas.

In the present flash survey, two-thirds of the respondents admitted that they might change priorities during this sanitary situation and more than a third did not think that a return to normality would happen before six months. These results are in contrast with the reduced acceptability of the cancer care deferral, but they show that, in spite of the lack of explicit knowledge, surgical oncologists remain highly adaptable to change. In Megginson’s words “According to Darwin’s Origin of Species, it is not the most intellectual of the species that survives; it is not the strongest that survives; but the species that survives is the one that is able best to adapt and adjust to the changing environment in which it finds itself ” [40].

The present survey benefitted from a high rate of Japanese respondents. Their availability to complete the survey is probably linked to the high influence of JSCO. Several high-quality Western organizations supported this survey but their area of influence is probably more heterogenous. The earlier phase in the management of COVID-19 at the time of the survey and the significantly lower percentage of Japanese surgical oncologists that had to alter cancer care explain lower acceptance of containment measures but probably should not be considered as the sole influencing factor as other cultural and social confounding variable were not taken into consideration by the present survey.

This flash survey can only picture the current situation. Attitudes of responders are likely to change during the pandemic as two-thirds of them confirmed. The surveyed sample of surgical oncologists was not specifically selected (biased) but is not representative neither due to the voluntary character. Response for most of the questions depended on many factors such as country of origin, setting, experienced phase of the pandemic, timing of response to the survey, but also general attitude, personality and values of the responders.

## Conclusions

Surgical oncologists have widely embraced the consequences of prioritizing the principle of “maximizing the benefits”, and strong and long-lasting support for lock-down measures aiming to save lives as top guiding principle. The pandemic forced surgical oncologist to accept the inacceptable, namely to modify best cancer care, probably for a prolonged period. To solve this dilemma, alternative strategies need not only to be developed but also to be monitored carefully to guarantee equipoise.
