# Supplementary material for: Priorities, actions and risks in the COVID-19 pandemic: a flash SoMe survey among surgical oncologists
Source: Pleura Peritoneum. 2021 Jan 25;6(1):7–12. doi: 10.1515/pp-2020-0142 (PMC8223800; doi:10.1515/pp-2020-0142)

**Online appendix 1** Survey

**Priorities, actions and risks in the COVID-19 pandemic: a flash SoMe survey among surgical oncologists**

Delia Cortés Guiral, Olivia Sgarbura, Mohammad Alyami, Fabian Grass, Martin Hübner

Dear colleagues:

The COVID-19 pandemic changed our professional and private lives within only a couple of weeks and the duration is uncertain. Radical measures needed to be taken in most countries to (I) contain infectious spread and (II) to increase/shift resources from most domains to emergency units and intensive care aiming to avoid a collapse of health care systems, exhaustion of care takers and to minimize the number of avoidable COVID-19 deaths due to insufficient resources.

The general principle of patient care “primum not nocere” is challenged in COVID-19 times, as most of the measures come at a price and medical, economic and socio-cultural consequences are tremendous but yet of unknown extent. For example, a compromise of cancer care is already reality in this early phase which risks to be only the beginning of a long and painful road!

This flash survey aims to open a differentiated discussion of values, priorities and necessary measures to cope with COVID-19 without jeopardizing cancer care and the economic and social fundaments of our societies.

Your answers are extremely valuable, this survey takes 5-7 min to be filled, we do appreciate your collaboration

**Demographic data:**

**Your country:**

**Your center is:**

1. An academic hospital
2. A Cancer Center
3. A private hospital
4. A local/regional public hospital

**You are:**

1. General surgeon dealing with cancer
2. Surgical oncologist

**You are super specialized in one of the following (please choose max 3):**

1. Colorectal cancer
2. Upper GI cancer
3. HPB cancer
4. Gynecological surgical oncologist
5. Breast surgical oncologist
6. Thoracic surgical oncologist
7. Peritoneal malignancies specialist
8. Not super specialized

**Experience level:**

1. Resident
2. Fellow
3. Consultant
4. **Guiding principles**

Please rank the following values according to their priority:

1 Save the most lives

2 Save the most life-years

3 Avoid collapse of the health care system

4 Protect health care workers

5 Maintain normal socio-cultural life, including schools/universities

6 Limit economic consequences including recession, bankruptcy, and unemployment

Are your priorities likely to change upon duration of the crisis?

Yes No

1. **Measures of containment**

Please rate your support for the following measures of containment on a scale from …

_____________________________________________________________

0 1 2 3 4 5 6 7 8 9 10

Not justified Inevitable

… and declare the acceptable duration for these measures by weighing expected positive effects but also negative consequences.

2 weeks 6 weeks 3 months as long as needed

1. Canceling of large public events like medical meetings and sports events
2. Prohibition of traveling and closing of borders
3. Closure of schools and universities
4. Lock-down of social and economic life
5. Handy tracking (or similar methods) to identify and sanction dissenters
6. **Shifting resources**

Please rate your support for the following actions to shift resources on a scale from …

_____________________________________________________________

0 1 2 3 4 5 6 7 8 9 10

Not justified Inevitable

… and declare the acceptable duration for these measures by weighing expected positive effects but also negative consequences.

2 weeks 6 weeks 3 months as long as needed

1. Shut-down of elective surgery program
2. Deferring or modification of cancer care

Have you already changed your strategies during the current crisis?

Yes No

Please describe shortly:

1. **Perspective**

Please give us your estimate for the duration of COVID-19 crisis and measures in your country:

1. Back to normal in sociocultural life
2. Back to normal in cancer care
3. Back to your normal surgical practice
4. Back to medical and surgical meetings

6 weeks 3 months 6months 1 year or longer

1. **Current management**

Please tell us about the specific management for cancer patients requiring surgical treatment in your country/hospital

1. Surgical treatment for cancer patients in covid-free centers
2. Surgical treatment is delayed but already scheduled within the next 3 months for all patients
3. Cancellation (postoponed without new appointment for surgery) of most oncologic surgeries
4. starting or prolonging the new-adjuvant therapy
5. Other strategies please describe shortly

Thank you for your collaboration

**Online appendix 2 Demographics of the responders**

1. **Country of origin**
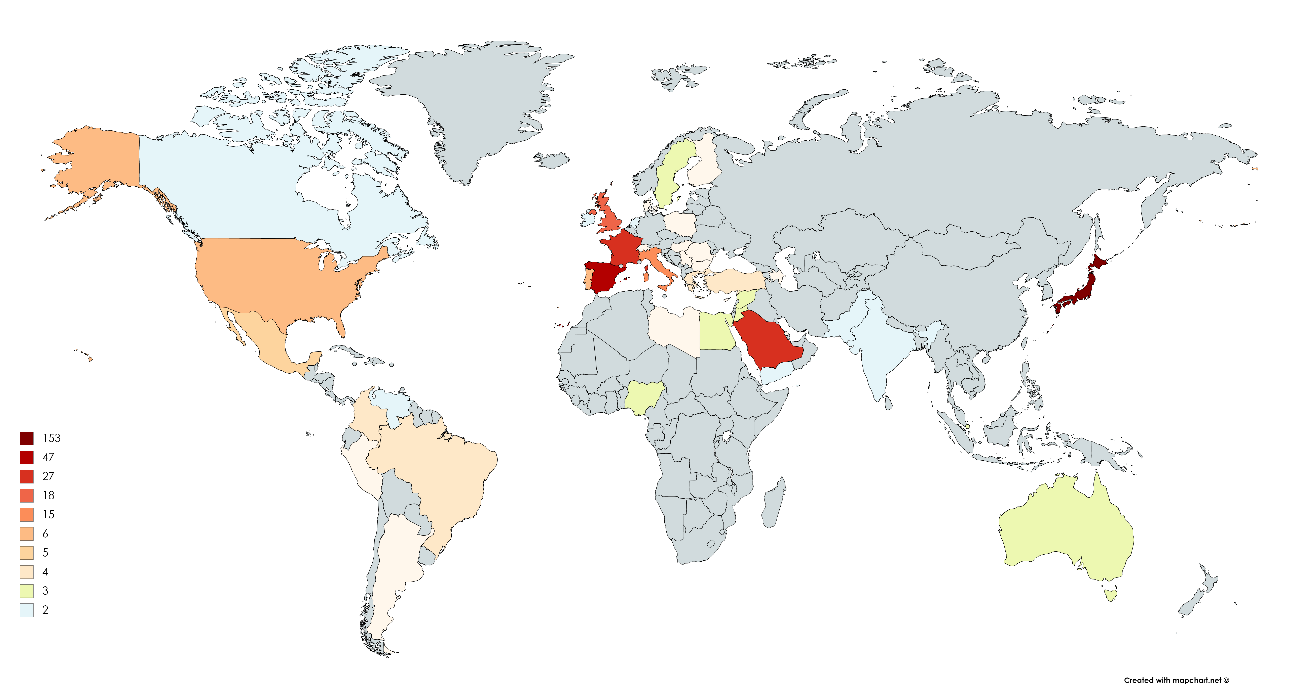

2. **Hospital setting**
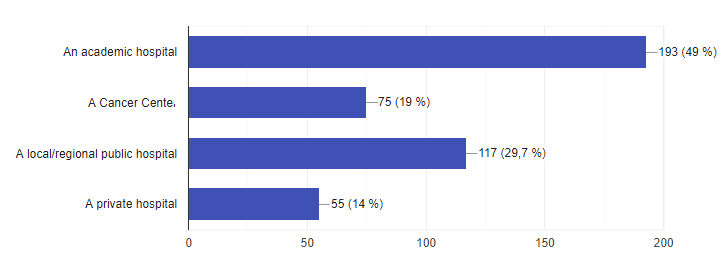

3. **Main activity**

**
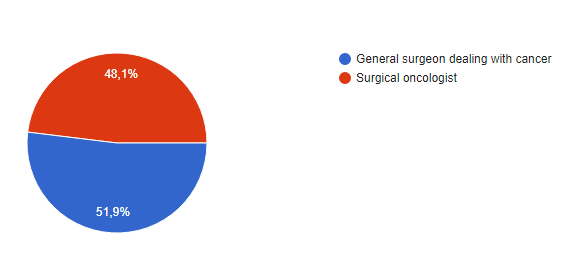
**

1. **Prevalence of sub-specialty**

**
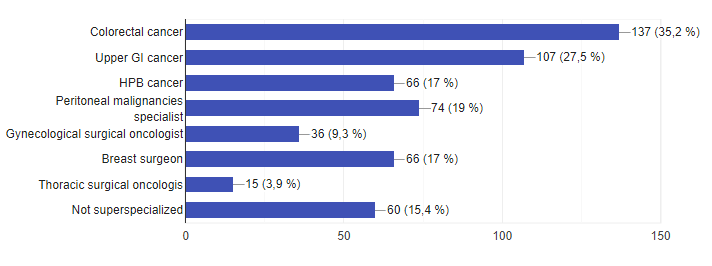
**

1. **Level of experience / position**


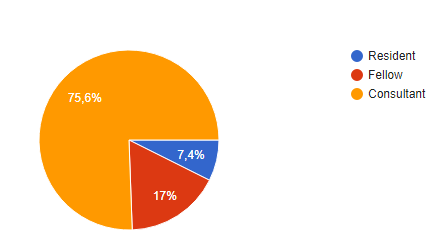

Supplement: Supplementary file 1 [file pp-06-20200142-s001.docx]
